# Unresolved Issues in RNA Therapeutics in Vascular Diseases With a Focus on Aneurysm Disease

**DOI:** 10.3389/fcvm.2021.571076

**Published:** 2021-04-15

**Authors:** Isabel N. Schellinger, Angelika R. Dannert, Karin Mattern, Uwe Raaz, Philip S. Tsao

**Affiliations:** ^1^Department of Cardiology and Pneumology, Heart Center at the University Medical Center Göttingen, Göttingen, Germany; ^2^German Center for Cardiovascular Research (DZHK) e.V., Partner Site Göttingen, Göttingen, Germany; ^3^Department for Endocrinology, Nephrology and Rheumatology, University Medical Center Leipzig, University of Leipzig, Leipzig, Germany; ^4^Department for Angiology, University Medical Center Leipzig, University of Leipzig, Leipzig, Germany; ^5^Division of Cardiovascular Medicine, Stanford University School of Medicine, Stanford, CA, United States; ^6^Veteran Affairs (VA) Palo Alto Health Care System, Palo Alto, CA, United States

**Keywords:** innovation, RNA, therapeutics, cardiovascular medicine, epigenetics, non-coding RNAs, abdominal aortic aneurysm

## Abstract

New technologies have greatly shaped the scientific and medical landscape within the last years. The unprecedented expansion of data and information on RNA biology has led to the discovery of new RNA classes with unique functions and unexpected modifications. Today, the biggest challenge is to transfer the large number of findings in basic RNA biology into corresponding clinical RNA-based therapeutics. Lately, this research begins to yield positive outcomes. RNA drugs advance to the final phases of clinical trials or even receive FDA approval. Furthermore, the introduction of the RNA-guided gene-editing technology CRISPR and advances in the delivery of messenger RNAs have triggered a major progression in the field of RNA-therapeutics. Especially short interfering RNAs and antisense oligonucleotides are promising examples for novel categories of therapeutics. However, several issues need to be addressed including intracellular delivery, toxicity, and immune responses before utilizing RNAs in a clinical setting. In this review, we provide an overview on opportunities and challenges for clinical translation of RNA-based therapeutics, with an emphasis on advances in novel delivery technologies and abdominal aortic aneurysm disease where non-coding RNAs have been shown to play a crucial regulatory role.

## Importance of Review

We believe that through the hereby provided review, we can give the interested readership a thoughtfully cured overview regarding the following topics:

1. A broad review on the ever-growing spectrum of RNAs with a focus on non-coding RNAs (including small and long non-coding RNAs).
2. An introduction to a widespread range of translational methods to apply RNAs as therapeutics (including nanocarriers and extracellular vesicles).
3. A comprehensive insight into challenges of translation in the field of RNAs.
4. Instructive examples where the mentioned challenges have been accepted and first results have been reached (including atherosclerosis and aneurysm disease).

## Introduction

Abdominal aortic aneurysm (AAA) is a common disease and defined as an abnormal focal dilation of the infrarenal aorta over 50% of the normal diameter (1). The normal diameter dependents on various factors such as age, gender, and body composition (2). Typically, an infrarenal aortic diameter ≥3.0 cm is considered aneurysmal (3). AAAs are subjected to treatment when the diameter reaches >5.5 cm, if rapid progression occurs (>0.5 cm/year) or if the AAA becomes symptomatic (4). Yet, AAAs are considered ticking time bombs as most patients remain asymptomatic until rupture. In case of rupture, bleeding is often fatal (5).

Factors increasing the risk for AAA development include smoking, male sex, and age >65 years (5). Interestingly the risk of AAA rupture is higher in females, although a smaller percentage of women generally suffer from AAA (6).

When a large pulsatile abdominal mass is found during physical examination, imaging is required to confirm that an aneurysm is present. Usually, abdominal ultrasound is performed to diagnose AAA (7) and lately ultrasound screening programs have been implemented in many countries of the European Union and in the USA to promote early disease diagnosis (8, 9).

Up to date, no pharmacological agent is available to prevent or treat AAA development and progression. Current therapies are limited to open surgical or interventional stent-graft based techniques. Unfortunately, these treatment options hold a high risk for procedural complications and are therefore reserved for larger aneurysms with a diameter >5.5 cm (4). Mortality regarding surgical treatment may be up to 5%, while endovascular repair displays endoleaks, stent graft migration and continuing AAA growth that may lead to secondary interventions or prostheses explanation (10).

On a molecular level, AAA pathology is characterized by smooth muscle cell (SMC) apoptosis, vascular inflammation, and extra cellular matrix (ECM) remodeling (11). Over the last couple of years, research has demonstrated that non-coding RNAs are significant modulators of these AAA-driving processes (12, 13). Thus, innovative RNA-targeting approaches hold great promise for revolutionizing clinical treatment of AAA disease.

## RNAs

When discussing the clinical potential of novel RNA-based approaches, there are several therapeutic categories that need to be distinguished. Most obviously, RNAs can serve as direct therapeutic targets to modify disease (14). Additionally, RNAs can act as mediators to indirectly achieve differential modulation of downstream regulatory networks. Moreover, RNAs can be used as tools for therapeutic intervention. In particular, short interfering RNA (siRNA) can be used to alter mRNA levels of particular target genes (14).

As of 2020, 10 nucleic acid based drugs have gained FDA-approval, of which two are implicated in cardiovascular conditions. Mipomersen, a gapmeR targeting ApoB, has been approved for homozygous familial hypercholesterolemia. The agent aids to reduce cardiovascular disease risk by reducing low-density lipoprotein (LDL) cholesterol levels. Defibrotide is a polydisperse oligo-nucleotide purified from porcine intestinal mucosa that has been approved as a drug against severe hepatic veno-occlusive disease, where it acts in a complex sequence-independent mechanism on fibrosis, angiogenesis, apoptosis, and aggregation of thrombocytes (15, 16). Other nucleotide-based therapeutic approaches are currently being tested in clinical trials (17, 18). So far, no nucleic acic based drug has been approved for treatment of aneurysmal disease.

### Small Non-coding RNAs

**Figure 1:**
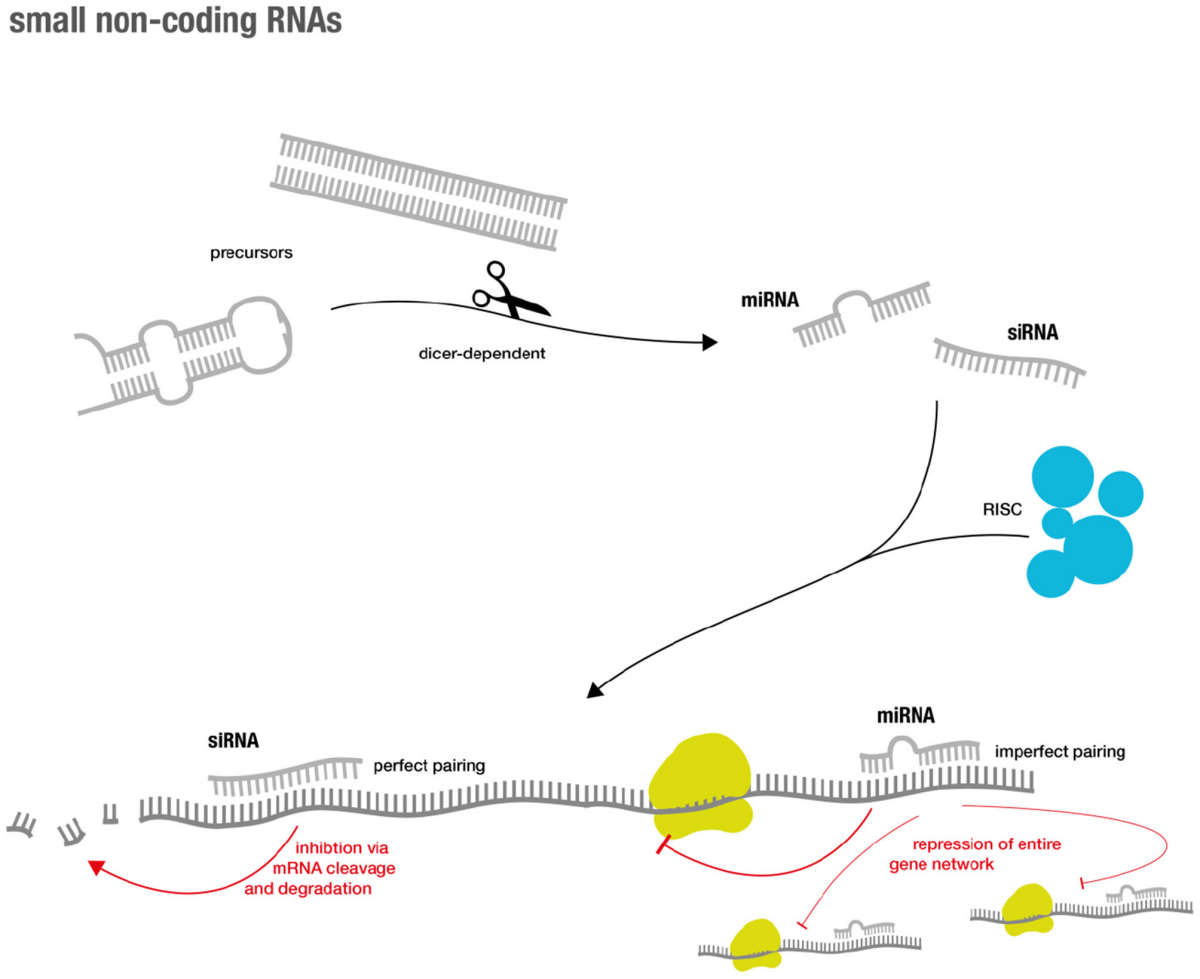
Overview over small RNA genesis and mode of action.

During the last years of the RNA revolution, small non-coding RNA molecules that are only ~20–30 nucleotides long, have emerged as important regulators of the eukaryotic genome (for overview see Figure 1). Two major classes are short interfering RNAs (siRNAs) and microRNAs (miRs). Traditionally, it was believed that siRNAs only originate from exogenous sources (e.g., viruses) while miRs are of endogenous origin (19). However, extensive research showed that this distinction was rather artificial (20).

Both classes generally inhibit gene expression and their effector functions are commonly summarized as RNA silencing. The latter is achieved via a complex succession of molecular sequences. In short, precursors (such as double-stranded RNA (dsRNA) and hairpin precursors) of both classes are processed via a Dicer-dependent mechanism that then enter the silencing machinery of the RNA-induced silencing complex (RISC) in their single-stranded form. The mRNAs of interest are targeted via base pairing of the small RNA component. This eventually results in sequence specific gene silencing (19). Perfect base-pair matching between the siRNA strand and the target mRNA is required for cleavage and subsequent degradation, while imperfect base-pair matching can result in miR-like repression (19, 21). The latter accounts for advantages and challenges in miR-based therapeutics. As miRNAs not only target one specific gene, but instead regulate entire functional gene networks (22), they might be able to address endogenous redundancy or compensatory mechanisms that cause difficulties for classical drugs (23). However, the broad regulation via miRs might also lead to unexpected and unwanted off-target effects (24).

Fire and Mello were the first to proof that experimental introduction of dsRNAs into a cell can be used to synthetically interfere with the function of an endogenous gene (25). Today, the RNA interference (RNAi) technology holds great promise to silence “non-druggable” targets that cannot be addressed using small-molecules, monoclonal antibodies, or other conventional therapeutic approaches (26).

### Long Non-coding RNAs

On the opposite site of the non-coding RNA spectrum, we find so called long non-coding RNAs (lncRNAs) (27). These comprise a diverse class of RNA molecules that do not code for proteins and that consist of >200 nucleotides and that are frequently polyadenylated (28).

Some lncRNAs are pseudogenic, copies of coding genes harboring mutations rendering them non-coding (29). Many lncRNAs overlap coding genes or are described as intergenic [known as long intergenic ncRNAs (lincRNAs) (30)]. But they also share many features with coding transcripts such as the occurrence of introns, the existence of splice variants and the presence of epigenetic marks indicating differential expression (29, 31). Additionally—without the need for translation—ncRNA expression is highly dynamic and can be rapidly up- or down-regulated to modulate gene expression. Their ability to fold into complex secondary structures also provides them with flexibility to interact with various substrates (such as proteins, RNAs, and DNAs) in a highly-specific manner (28, 32).

Regarding target sites, it is possible to distinguish between cis- and trans-acting lncRNAs. Cis-acting lncRNAs control the expression of genes in close neighborhood, whereas trans-acting lncRNAs function from a distance (28).

Regarding lncRNA location, one can differentiate between cytoplasmatic lncRNAs that modulate translational control and nuclear lncRNAs that guide chromatin modifiers to specific genomic loci (33–35). The latter usually results in DNA and histone modifications that yield inhibitory heterochromatin and transcriptional repression (34).

However, transcriptional activation has also been reported especially via activation of specific enhancer regions or by “sponging” miRs. LncRNAs that act as negative regulators of miRs and thereby achieve mRNA upregulation function as competing endogenous RNAs (ceRNAs) (36, 37). Linear ceRNAs have a short half-life that allow for quick control over sponge activity, while circular sponges—such as circRNAs—have much greater stability due to their resistance against endonuclease degradation (38).

**Figure 2:**
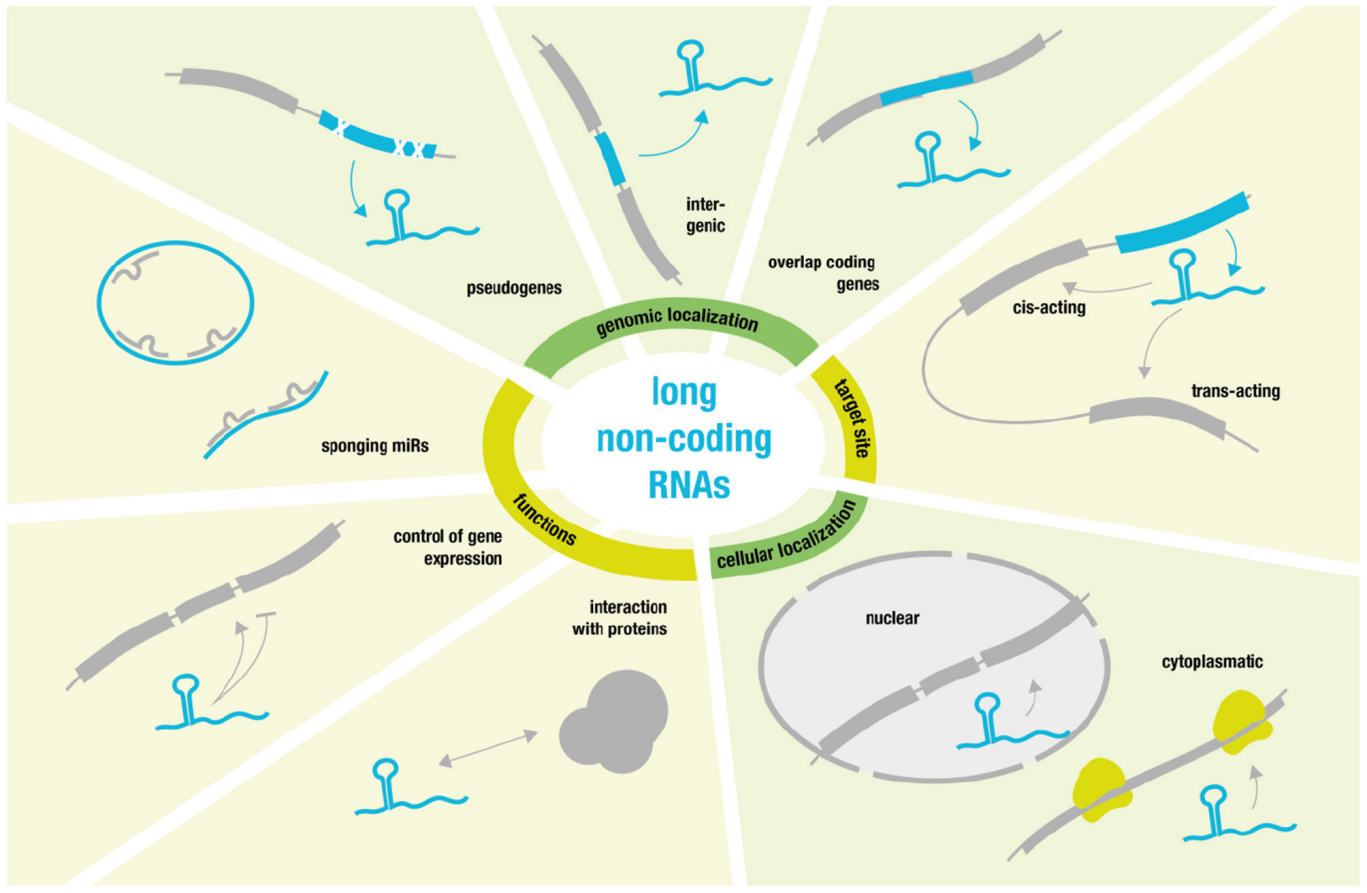
Overview over long non-coding RNA function and behavior.

Moreover, some of the best-characterized lncRNAs are involved in processes like X chromosomal inactivation (XIST) (39) and genomic imprinting (H19) (40). Yet, much more research will be necessary to characterize the many diverse functions and modes of action related to lncRNAs [for more detailed review see (28) and for overview see Figure 2].

In the context of aneurysm disease H19 has been identified to boost aneurysm progression (41).

## RNAs as Therapeutics

Using RNAs for therapeutic approaches has many advantages due to their unique biology. Targeting and editing the undruggable human genome, modulating gene expression and altering epigenetic processes are just some of the many opportunities (26).

However, current RNA-related methods are restraint by a couple of significant factors. RNAs are predisposed to endonuclease degradation (26), can cause adverse immunological reactions (42), and their large size and negative charge prevent them from passively crossing the cell membrane's lipid bilayer (43). Generally, RNA molecules are taken up by endocytosis and remain trapped inside the endosome, but outside the cytoplasm and nucleus (44). During the last decade, significant research has therefore focused on addressing the many challenges associated with RNA-based delivery.

Today, single-stranded antisense oligonucleotides (ASOs) usually contain a phophorythioate backbone replacing the early phophodiester bonds. This results in increased stability and hydrophobicity, thereby facilitating cellular uptake, enhancing bioavailability, and improving pharmacokinetics by binding serum proteins like albumin (45, 46).

ASOs usually function via cleavage of a formed mRNA:ASO duplex by a recognition enzyme termed RNase H (47). One setback in this regard is the reduced binding affinity of phosphorothiorate ASOs toward the mRNA target (48). Binding affinity can be improved by incorporating 2′ modifications [such as 2′-O-methyl (2′-OMe), 2′-O-methoxyethyl (2′-MOE), and 2′-Fluoro (2′-F)] (49). These modifications however reduce nuclease degradation (50).

Naked (unconjugated) ASOs—just like other RNAs—enter the cell via endocytosis (51). Yet, they have the ability to slowly cross the lipid bilayer and escape into the cytoplasm and nucleus (gymnosis) due to their hydrophobic characteristics (45, 46, 52). The latter render ASOs independent of additional delivery agents (53).

The specific delivery to many cell and tissue types remains difficult via current ASO methods. In this context, conjugating cell-type-specific targeting domains to ASOs might help to overcome this huge challenge. Successful examples for the mentioned approach include the N-acetylgalactosamine (GalNAc) ligand that is used for targeted ASO delivery to hepatocytes via the hepatocyte-specific asialoglycoprotein receptor (54, 55).

ASOs that include a 2′,4′-O-methylene link between two joint rings (rendering the molecule bicyclic) are known as locked nucleic acids (LNAs). LNAs have an overall enhanced resistance toward degradation by 3′-exonucleases and show a great affinity to their mRNA target. The latter decreasing molecule length and improving delivery (56, 57).

LNAs can be further divided into two main groups: mixmers and gapmers. Mixmers contain LNAs and DNA nucleosides that are spread throughout the oligonucleotide sequence. Mixmers block protein translation but are not able to support mRNA cleavage (48). Conversely, gapmers comprise a central segment (gap) that is flanked by two LNA segments at both ends of the oligonucleotide and inhibit mRNA expression (by recruiting RNase H) and protein translation alike (58). One prime example for a novel FDA-approved RNA drug is developed as a gapmer. Mipomersen is directed against ApoB to counteract homozygous familial hypercholesterolemia (59).

Another subclass of ASOs are so called “antagomirs” or “anti-miRs.” These terms are used to describe single-stranded oligonucleotides that prevent miRs from exerting their inhibitory effect on their respective target mRNAs (60). Antagomirs are able to reach many different tissues without crossing the blood-brain barrier and their effects last at least 3 weeks after intravenous injection (60). However, their way of functioning has not been completely understood, yet. Chemically, antagomirs show a close relation to their double-stranded siRNA counterpart (61).

## Double-Stranded siRNAs

ASOs and siRNAs show prominent differences regarding their mode of action. As described above, ASOs bind their mRNA target in a direct and unassisted manner, therefore permitting for various chemical modifications. SiRNAs rely on supporting molecules such as the dicer enzyme and the RNA**-**induced silencing complex (RISC) (62). This dependency restricts the extent of possible chemical modifications greatly (63, 64).

Introduction of phosphorothioates has improved siRNA stability and shortening of the siRNA double strand has reduced overall charge for enhanced local delivery (65–67). Other modifications include a “Trojan horse approach” via short-interfering ribonucleic neutral (siRNN) molecules that contain a phosphotriester backbone. Initially, siRNNs mimic a neutrally charged protein surface. After entering the cell, they are cleaved into negatively charged phosphodiester siRNAs that induce a RNAi response (68). Despite all modifications, siRNAs remain macromolecules that cannot cross the cell's lipid bilayer. Thus, siRNAs depend on aiding delivery agents (43).

Lipid nanoparticles (LNPs) and synthetic nanoparticles were early attempts to master the siRNA delivery dilemma. Both systems were originally used for DNA-mediated gene therapy and required optimization for a siRNA approach (69, 70). But supplementing additional components to LNPs increases toxicity and decreases solubility, thus leading the RNAi field away from large LNPs to smaller, more tailored methods (44, 71).

When it comes to hepatic targeting, GalNac conjugation—similar to the ASOs approach described in the section above—has been successfully applied (72). For extra-hepatic delivery, antibodies hold great promise for a more specific delivery approach (73). However, difficulties regarding the antibody-RNAi conjugate's expression, aggregation, and activity need to be addressed, before entering the clinical trial stage (74).

### CRISPR-Cas9 Directed Approaches

A novel and utmost elegant way of using RNAs for therapeutic modulation is genome engineering via Clustered Regulatory Interspaced Short Palindromic Repeats (CRISPR) and the CRISPR-associated Proteins (Cas) proteins. Originally, CRISPR/Cas is a bacterial defense mechanism that prokaryotes use against viral and plasmid intruders. Prokaryotes integrate DNA sequences that match past intruders into their genome as part of their adaptive immune response. This intruder library or memory helps the organism to recognize and defend itself during future attacks (75).

Today, the CRISPR/Cas9 type II system is commonly used in the research set-up. There have been other nuclease-based systems that contain genome editing capabilities but CRISPR is highly user friendly due to its simplicity and flexibility (76). In short, the Cas9 nuclease is guided to the target sequence adjacent to a protospacer-adjacent motif (PAM) by a single guide RNA (sgRNA). There, a double-strand break is produced that can be repaired by non-homologous end joining (77). The latter usually leads to insertions or deletions resulting in loss of function (knock-out). However, gain of function (knock-in) can also been achieved via exogenous donor DNAs.

Just as with the above described techniques there are several delivery hurdles to take when transferring CRISPR/Cas9 into a clinical context. Both Cas9 and the sgRNA are charged macromolecules that are difficult to introduce into the cell's nucleus (78).

Other concerns include off-target effects that could be reduced by adapting the sgRNA design (79, 80). Design algorithms have already been implemented focusing on the 5' sequence of the sgRNA to maximize on-target action (81). Another reason for off-target effects are unintended high levels of Cas9 nuclease associated with unspecific cleavage (82). There are different strategies to control the activity of Cas9 such as generation of Tet-controlled promoters (83), fusion of Cas9 with an estrogen receptor domain (84, 85) or usage of light-responsive elements (86) to control Cas9 activity.

Some of the main questions linked to genome engineering via CRISPR/Cas are of ethical nature as editing a patient's genome would cause a life-long alteration of the human “code” (87). At the moment, the CRISPR/Cas method is still at a very early step of its development and the next years and decades will show how to apply this exciting, new system to achieve the best possible outcome for every single patient.

## Delivery Systems for RNAs

**Figure 3:**
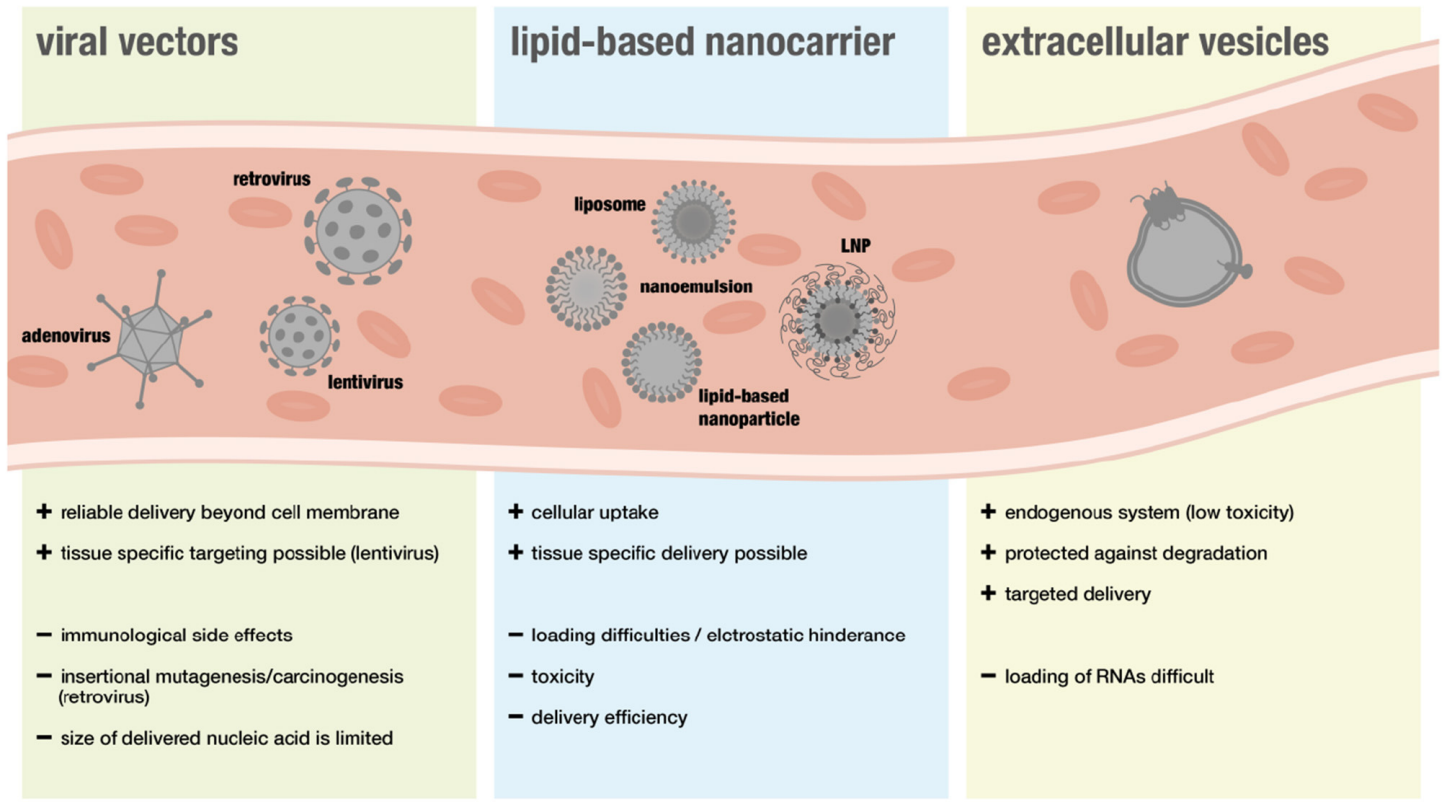
Overview over different delivery system in the context of RNA therapeutics.

The following section will give an overview on different delivery systems used for RNA therapy including viral and non-viral vectors (for overview see Figure 3).

### Viral Delivery

Viral vectors are considered a suitable approach for gene therapy because they can deliver nucleic acids easily beyond the cell membrane. However, safety concerns have been limiting translational efforts. In particular immunological side effects and insertional mutagenesis leading to cancer development raised concerns (88). Furthermore, some people show immunity toward specific viral vectors and the size of nucleic acids that can be transported via viral systems is more restricted than with non-viral systems (89).

Retroviruses were among the first viral vectors used for cell transfection with RNA-expressing plasmids. These viruses provided great results *in vitro*, while they were linked to safety concerns and limited efficiency *in vivo*—especially in the human setting (90). Retroviruses integrate their viral genome into the host genome for viral replication. This mode of action can cause insertional mutagenesis and even carcinogenesis (91, 92). Furthermore, retroviruses need replicating cells for RNA therapy. However, most mammalian cells are in resting state and not actively replicating. This causes difficulties for retroviruses in reaching the cell type of interest (93).

Thus, lentiviruses, a subclass of retroviruses, have emerged as appealing vectors for *in vivo* application. While replication competent lentiviruses—virus particles capable of infecting cells and replicating to produce additional infectious particles—remain a safety concern, compared to other retroviruses, lentiviruses have reduced risk of insertional mutagenesis (94) and are able to transfect non-dividing cells (95). Additionally, they are less immunogenic than adenoviral vectors (96) and can be used for tissue-specific targeting (97). The latter can be achieved via integration of envelope material from other viruses. Experiments with murine embryos showed that, e.g., Mokola or Ebola virus glycoprotein can confer certain tissue tropism toward heart and skeletal muscle cells (MVG and EVG) (98).

Adenoviral vectors are a popular type of viral vector for gene therapy trials that have been widely studied (99). Different to other viral vectors the genetic information does not integrate into the host cell's genome but persists in episomal form in the nucleus (100). On one side this reduces the risk for viral DNA integration into the host cell's genome, on the other side this leads to more transient effects (100). To achieve long-term transfection *in vivo*, all viral coding sequences were removed and immune responses against transduced cells limited (101). Nevertheless, the high (also dose dependent) immunogenicity and hepatotoxcity of certain adenoviral proteins remains a concern (102, 103). Traditionally, human adenovirus are classified in unevenly prevalent serotypes based on immune cross-reactivity. Some of the immunity is broadly spread thereby diminishing the potency of adenoviral vectors. To bypass this challenge, adenoviral vectors are mostly derived from rare serotypes. Other classifications are based on sequence homology for the identification of new adenoviruses (104). Targeting specific cell populations, apart from the natural tropism of vectors, was achieved by specific re-targeting approaches leading to an expansion in transduction of specific cell populations (105).

Through upgraded vector design the safety of the delivery systems could be improved [as reviewed in (88, 99)] and today more than two thirds of all gene therapeutically clinical trials registered until the end of 2018 (a total amount of 2,600) have been conducted using viral vectors (17).

Some viral vector based therapies have been cleared by the FDA including adenovirus associated vectors and many clinical trials are active.

### Lipid-Based Nanocarriers

Although many advances have been made regarding safety, viral vectors are still associated with a higher risk of triggering adverse immunogenic responses. Thus, non-viral nanocarriers have been widely favored when it comes to RNA-delivery. Lipid-based nanocarriers can enable cellular uptake of RNAs, as their natural structure is ideal when it comes to interactions with the cellular phospholipid membrane (106).

Nanoemulsions consist of two immiscible liquids that are mixed by using specific surfactants to form a single phase (107). Although it is relatively easy to mix several compounds into a single nanoemulison formulation, this method shows some challenges when applied to the RNA field. One advantage of nanoemulsions is the relative ease to mix multiple ingredients in a single formulation. By convention, nano-emulsions are made of negatively charged ingredients. This will make it problematic to trap RNA molecules within a nanoemulsion (108). Furthermore, keeping the nanoemulsion's fine lipid droplets small and uniform while stored after inserting the quite large and hydrophilic RNA molecules (108).

Another form of nanocarriers are lipid-based nanoparticles. They encounter difficulties regarding RNA delivery due to their lipophilic core that does not match the hydrophilic, poly-anionic RNA molecules. However, this structure allows to encapsulate lipophilic drugs into the core and coat the RNA on the nanoparticle surface thereby creating unique complexes of high translational potential (109).

Liposomes are easily prepared and consist of an aqueous core surrounded by a phospholipid bilayer (110). Tissue-specific delivery and extended circulation time can be achieved via functionalization (e.g., PEG-lipids) (111, 112). The aqueous core renders liposomes suitable for loading hydrophilic and ionic drug molecules, distinguishing them from other lipid-based systems that are usually loaded with lipophilic compounds (110). Subsequently, positively charged (cationic) liposomes can readily encapsulate RNAs and are therefore considered most suitable when it comes to RNA delivery. Unfortunately, cationic lipids are also the main driver of toxicity regarding lipid-based RNA nanocarriers (113). Alteration strategies [detailed review in (114, 115)] have been implemented to improve both transfection performance and cytotoxicity. Phospholipid molecules typically consist of three main components: a cationic head, a hydrophobic hydrocarbon backbone and a linker region (116). Each component can be modified to achieve certain molecule properties. DOTAP that is considered today's gold standard for RNA transfection of cationic liposomes has a linker consisting of biodegradable ester bonds thereby limiting toxic effects (117, 118).

In terms of clinical translation, LNPs (lipid nanoparticles) represent the most successful type of RNA nanocarrier (119). LNPs are ~120 nm in diameter or less, composed of neutral lipids, cationic lipids, and PEG-conjugated lipids and form by spontaneous vesicle formation (120). LNPs have integrated many of the mentioned design strategies to overcome multiple challenges associated with RNA delivery, including limited *in vivo* circulation, inefficient transfection, and unacceptable toxicity (120).

### Extracellular Vesicles

A new type of RNA-delivery for therapeutic purposes are extracellular vesicles (EVs) that have recently been discovered to be endogenous RNA carriers. EVs are released by cells and serve as means of intracellular communication. Since cells use miR-loaeded EVs to regulate gene expression in other cells and different sorts of RNAs, including lncRNAs, were found to be presented in these vesicles, they might represent a safer and more effective method of RNA-delivery (121–126).

Interestingly, there seems to be a clear pattern when it comes to RNA vesicle packaging. Certain sequence motifs are favorably integrated into EVs (127). Moreover, there is evidence, that proteins of the RISC complex are involved in the packaging process since they were detected inside of the EVs as well (128).

One major advantage of RNAs that are loaded into EVs is that they are protected from degradation by extracellular RNases (129). EVs serving as delivery systems are also considered superior with regards to targeted delivery and cytotoxic side effects. Yet, a major problem remains in loading the RNA therapeutics into the vesicles. Due to the negative charge of the RNAs, crossing the vesicle membrane represents a major challenge. Therefore, most studies investigating EVs as delivery tools are focused on siRNAs and miRs rather than lncRNAs due to their restricted size that facilitates vesicle packaging (126).

RNAs have successfully been loaded into EVs prior to isolation which raised concerns about the purity of the generated RNA-loaded vesicles. Consequently, several different mechanisms have been established to incorporate RNAs into EVs after isolation, including electroporation, sonication, and incubation with varying degree of success, as extensively reviewed in Jiang et al. (126).

## Off-Target Effects and Cross-Reactivity

**Figure 4:**
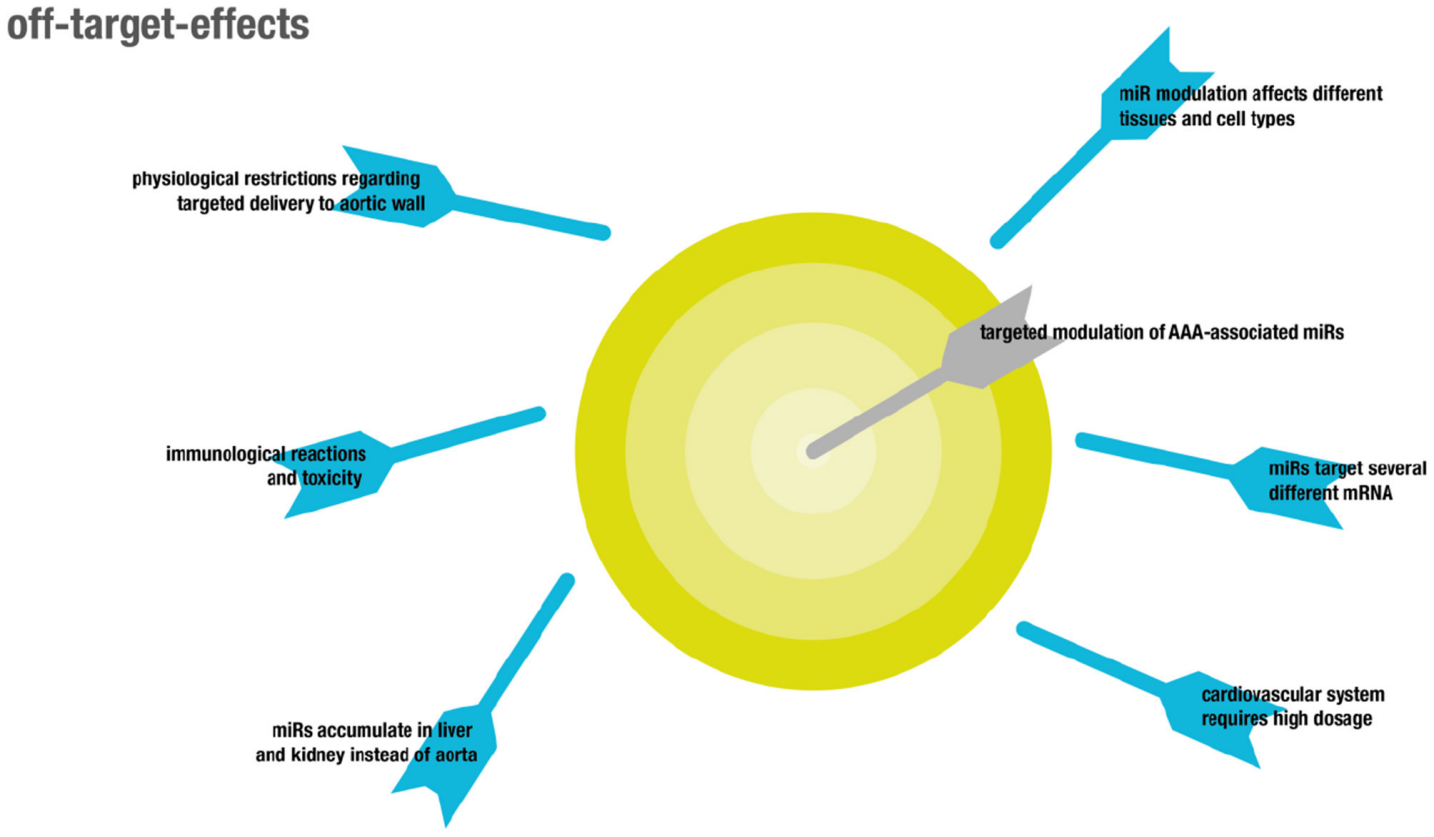
Overview showing off-target effects in the context of RNA therapeutics.

There are two major types of off-target effects that can be distinguished (for overview see Figure 4). On the one hand, off-target effects result from ubiquitous expression of targeted coding and non-coding RNAs in different tissues and cell types, resulting in unintended systemic treatment effects (130). On the other hand, off target effects may be due to the promiscuous mode of action of miRNAs that usually do not target one but several mRNAs (22, 131).

Although miRNAs were shown to target functional gene networks (23), unwanted off-target effects are likely to occur, due to inherently imperfect binding to mRNA sequences (24). In particular, some microRNAs relevant in the cardiovascular system might play differential roles in different pathologies, like cancer. For example, overexpression of miR-21 was shown to be protective in two models of AAA by influencing apoptosis and proliferation of vascular smooth muscle cells in the aortic wall via PTEN and AKT signaling, resulting in attenuated expansion of AAA (132). However, miR-21 is also well-established as an onco-miR with upregulated levels in several different kinds of solid and leukemic malignancies, likewise attributed to its regulatory function on PTEN (133).

Another example is miR-29b. While LNA-mediated inhibition of miR-29b increased fibrosis as indicated by collagen expression and thus resulted in attenuated growth of AAA, induction of fibrosis is pathological in other organs, which makes systemic delivery of miR29b inhibitors non-feasible (134). Targeted delivery to the aorta is complicated by the inherent feature of the aorta that any compound locally delivered to the blood will immediately be distributed throughout the entire circulation, which is in contrast to targeting other vascular structures like the coronary arteries (135). This process results in accumulation of the delivered compounds in highly perfused capillary beds in non-target organs like the liver. Adverse effects in general primarily affect both liver and kidneys, since exogenously administered RNAs preferentially accumulate in these organs (136). Moreover, both liver and kidney are responsible for clearance of naked RNA from the bloodstream, on the one hand through accumulation in the liver and on the other hand through glomerular filtration in the kidney (137). As a consequence, the first therapeutic approaches involving RNAs these days target hepatic diseases since therapeutic concentrations of RNA can be reached most easily in this organ. In particular, nanoparticles and cationic lipid-dependent systems strongly accumulate in the liver (138). Accumulation in other organs might be achieved by use of specific ligands binding to receptors on the target cells which was already shown for vascular targets (139). However, as already mentioned this might be difficult in the case of aortic aneurysms because receptors on relevant cell types like SMCs are not in direct contact with the blood stream. The dose commonly administered in animal models to target the cardiovascular system is about 3–10-fold higher than that used in animal models of liver pathologies. This increases toxicity as well as potential unspecific binding and thus off-target effects, consequentially narrowing the therapeutic window (140).

## RNA-Based Therapy in Vascular Diseases

### Non-coding RNAs in AAA

**Figure 5:**
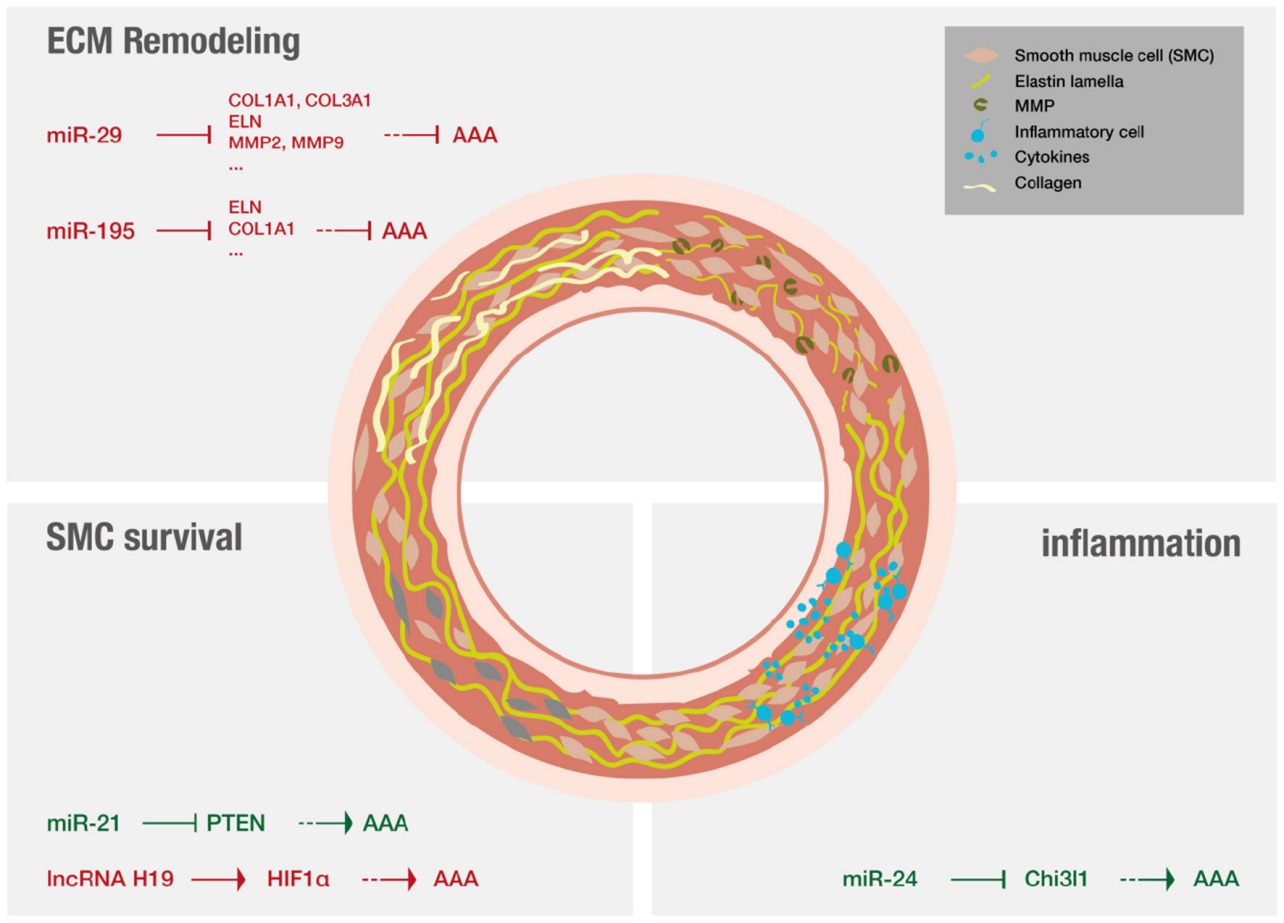
Non-coding RNAs that have been shown to play a major role in AAA disease.

AAA pathology is characterized by three distinct mechanisms on a molecular level (for overview see Figure 5): ECM remodeling, vascular inflammation and SMC apoptosis (11). Lately research has demonstrated that non-coding RNAs are significant modulators of these processes (12, 13).

MiRs are of special interest in a translational context as they are able to modify whole gene networks (23). Concerning *fibrosis* miR-29 has evolved as an important player (134, 141) and the effect of LNA-anti-miR directed against miR-29 on the formation of aneurysms was studied extensively. Mägdefessel et al. (134) found miR-29b to be significantly downregulated during murine AAA development as well as in human AAA tissue (134). The study found increased expression of several different collagen isoforms as wells as elastin upon treatment with LNA-anti-miR-29b in two different murine models of AAA, using either Angiotensin II infusion in apoE^−/−^ mice or porcine-pancreatic-elastase infusion in C57Bl/6 mice. In both models, LNA-anti-miR-29b treatment resulted in reduced formation of AAA (134). The opposite results were found when the mice were treated with miR-29b mimics. However, this study also demonstrated increased collagen mRNA levels in the heart, kidney, and liver upon systemic administration of anti-miR-29b, indicating off-target effects that demand for local delivery of the anti-miR-29b (134). In line with these results, another study evaluated the therapeutic potential of miR-29b in a mouse model of Marfan syndrome (142). LNA-anti-miR-29b was administered both prenatal and postnatally by retro-orbital injection. The investigators found that anti-miR-29b could reduce formation of AAA in the long-term if administered prior to formation of AAA but did not ameliorate the outcome of already formed AAA. Anti-miR29b treatment, if administered prenatally resulted in increased expression and decreased breakdown of elastin in the aorta, potentially attributed to decreased matrix-metalloproteinase-2 (MMP-2) activity and expression (142). Furthermore, collagen levels were found to be increased upon treatment in both studies (134, 142). The manipulation (either inhibiton or overexpression) of other miRs can also ameliorate AAA disease by increasing collagen content and reducing elastin fragmentation. Two examples are miR-181b that is effective by regulating macrophage timp3 expression and miR-126-5p that signals via ADAMTS-4, an enzyme that cleaves various ECM proteins (143, 144).

Regarding *inflammation* in the context of AAA, miR-24 was identified as key regulator in both established murine models as well as in human aortic tissue and plasma analysis (145). Inhibition of miR-24 showed enhanced AAA development, while upregulation of miR-24 ameliorated disease. It was further revealed that chitinase 3-like 1 (Chi3l1) is a major target of miR-24. *In vivo* and *in vitro* experiments additionally showed that Chi3l1 not only regulates cytokine synthesis and survival of macrophages but also promotes aortic smooth muscle cell migration and cytokine production, and stimulates adhesion molecule expression in vascular endothelial cells (145). A recent review focused on myeloic cells and aneurysm disease (146).

Other studies investigated RNA effects on *SMC survival*. MiR-21 levels were found to increase during AAA development in two different murine models, miR-21 overexpression was shown to increase SMC proliferation via reduction of phosphatase and tensin homolog (PTEN) protein, leading to increased phosphorylation and activation of AKT thus having protective effect on AAA development (132). The results could be confirmed using LNA-anti-miR-21 constructs that were applied systemically and resulted in increased apoptosis and decreased proliferation of SMCs, thus strongly aggravating AAA (132). Interestingly, miR-21 levels were shown to be increased in a nicotine-dependent manner. Upregulation of miR-21 in AAA as well as increased upregulation of miR-21 in AAA of smokers could be confirmed in human samples (132). Additionally, a recent study reported that inhibition of long non-coding RNA H19, utilizing LNA-GapmeRs *in vivo*, significantly limited aneurysm growth in established murine models by influencing SMC survival. This study proofed especially translational as it investigated similar patterns in human AAA tissue samples, and in a novel preclinical *LDLR*^−/−^ Yucatan mini-pig aneurysm model (41).

Zampetaki et al. (147) evaluated the effect of local as well as systemic delivery of antagomiR-195 on the development of aortic aneurysm in ApoE^−/−^ mice treated with Angiotensin II. AntagomiR-195 was found to de-repress aortic expression of elastin and other extracellular matrix genes after systemic application while antagomiR-29b mainly affected gene expression in the liver, thus already indicating potential off-target effects. The antagomiR-29b effect on the development of AAA was more pronounced than the effect of antagomiR-195 (147). The antagomiRs used were cholesterol-conjugated and delivered by peritoneal injection. For evaluation of local effects, an aortic isograft model using aorta incubated with cholesterol-bound LNA-antimiR-195 was used. Local delivery showed additional de-repressive effects on MMP expression. This study also evaluated the effect of antagomiR-195 on closely related miRs of the miR-15 family and did not find any off-target effects in that regard (147).

More evidence on vascular targeting of antagomiRs derives from studies on atherosclerosis and restenosis. Systemic antagormiR-92a delivery was already shown to be effective in downregulating vascular miR-92a and thereby leading to decreased atherosclerosis and endothelial dysfunction in *LDLR*^−/−^ mice that were fed a high-fat diet (148). This provides evidence, that systemic antagomiR delivery can successfully target the aorta. Notably, the measured effects in this study were higher at the aortic arch than at the thoracic aorta. Furthermore, effect of antagomiR-92a was not assessed in other organs (148). In another study, antagomiR-92a was shown to potentially reduce restenosis in a rat model of carotid artery balloon injury. While also this study implies successful delivery of the antagomiR to the vasculature after systemic administration, no off-target effect were evaluated either (149).

Despite limitations, different studies examining the effect of miR inhibition or stimulation by systemic mechanisms successfully detected positive effects on AAA development. Unfortunately some studies also found systemic side-effects (134, 142) that need to be even more carefully evaluated in the future when clinical translation wants to be achieved.

### Nanoparticles in Atherosclerosis

While targeted delivery of anti-miR to treat AAA using nanoparticles to our knowledge has not been reported yet, this approach has been investigated for the treatment of atherosclerosis.

One study exploited the fact that along with miR-712, disturbed flow in blood vessels also leads to the upregulation of vascular cell adhesion molecule 1 (VCAM-1) on endothelial cells. Coated cationic lipoparticles were modified using a ligand for the VCAM-1 internalizing sequence (139). This approach tackles different challenges in miR delivery. It increases transfection efficiency due to the presence of cationic lipids in the lipoparticles, while simultaneously reducing toxicity and aggregation due to a coat of neutral lipids. Furthermore, it enables targeted delivery to inflamed and early-stages of atherosclerotic vessels by providing a ligand that first binds VCAM-1 and then initiates its internalization. The study examined the effect of their nanoparticles on the development of atherosclerosis in a partial carotid ligation model in apoE^−/−^ mice. While leading to specific downregulation of miR-712 in endothelial cells of affected areas and prevention of atherosclerosis development, no impact on miR-712 expression was observed in other vascular regions or any other organ investigated. During *in vivo* experiments, no differences in blood proteins or blood cell counts were observed between treated and control mice. However, *in vitro* results showed increased C3a levels after treatment with targeted nanoparticles (139). Therefore, investigations of the immune response and consequences of chronic treatment remain issues that need to be addressed in this promising approach. As demonstrated in the study above, anti-miR-712 was undetectable in the aortic media (139).

With regards to treatment of AAA, targeted delivery will be more difficult, since a potential receptor on SMCs or fibroblasts will not be in direct contact with the systemic blood stream. However, recent studies show progress in side-specific administration (150).

### Microspheres in Ischemic Heart Disease

Another study used microspheres to deliver antagomiR-92 into the coronary arteries in a pig model of perfused acute myocardial infarction to limit post-infarct remodeling, dysfunction of vessel wall motion, and promote vessel growth (135). This was successfully accomplished by a single intracoronary injection of antagomiR-92 loaded microsphereses shortly after reperfusion. The antagomiR was designed as a LNA to increase stability in the blood, and did not have any ligands to mediate cell-specific delivery. After injection, the microspheres were shown to be present in the capillaries of the damaged area and were not detectable in other organs, thus demonstrating successful local delivery without major spill-out into the circulation (135). However, this also leads to the conclusion that this approach is not feasible for treatment of aortic aneurysms since the microspheres are likely to accumulate in the capillary beds of downstream organs, thus increasing off-site effects.

### Extracellular Vesicles in Cranial Aneurysm

To our knowledge, there is no study using extracellular vesicles to target RNA therapeutics to AAA. However, it was shown that when administering mesenchymal stem cell derived extracellular vesicles to a murine model of intracranial aneurysm the rate of aneurysm rupture was decreased (151). While the extracellular vesicles were detected in brain tissue, they were also present in spleen, liver, and lung of the investigated mice. Of interest, administration of the extracellular vesicles resulted in decreased mRNA levels of target genes like tumor necrosis factor α (TNFα), increased cyclooxygenase 2 (COX2), and E-prostanoid-4 mRNA levels, thus contributing to decreased aneurysmal rupture. Changed mRNA expression patterns might be indicative of miRNA as one of the effective agents within the extracellular vesicles in this study (151).

### Viral Vectors in AAA

Several studies investigating the role of different miRNAs employed viral vectors to investigate the effects on gene expression *in vivo*. For example, one study found miR-24 to be protective against AAA development using lentiviral vectors to deliver pre-miR-24 in two different murine models of AAA. In both models decreased aneurysm development was demonstrated. The results could be confirmed using lentiviral vectors expressing anti-miR-24 which led to aggravated aneurysm development (145). A more recent study demonstrated decreased AAA formation after infection with miR-145 containing lentivirus in an aneurysm model of Ang II infused ApoE^−/−^ mice. This therapeutic effect was mediated by miR-145-dependent downregulation of MMP-2 expression (152). In a different approach, bone-marrow derived leukocytes were infected with lentiviral vectors containing siRNA against C-C chemokine receptor type 2 (CCR-2) which is known to be involved in the initial pathogenesis of AAA as a part of the immunological degradation of ECM components (153). The transduced cells were delivered by bone marrow transplantation prior to Ang II infusion in an aneurysm model of Ang II infused ApoE^−/−^ mice. This siRNA approach significantly reduced the aneurysmal damage to the aortae in these mice (153).

### Medical Devices in AAA

Another possibility of local delivery that is particularly feasible for AAA (and clinical translation) are drug-eluting stents. This stent approach has been tested in a humanized myointimal hyperplasia model for in-stent restenosis using balloon-injured human internal mammary arteries that were transplanted into Nowett-nude rats (154). The miR under investigation for its therapeutic relevance regarding in-stent restenosis was miR-21. Due to substantial off-target effects in liver, lung, heart, and particularly kidney after systemic delivery of LNA-anti-miR-21, anti-miR-21 coated stents were tested. While the anti-miR eluting stents showed significantly reduced neointima formation compared to conventional stents, no off-target effects were observable, not even in the kidney that showed marked LNA-anti-miR accumulation after systemic application (154). With regard to AAA, these study demonstrates that local delivery of miR inhibitors to the vasculature is possible and is potentially advantageous over systemic delivery not only with regards to off-target effects, but also regarding treatment efficiency. Drug-eluting stents are a promising route of delivery to the thoracic and abdominal aorta.

In a pig model of acute ischemia and reperfusion injury, delivery of LNA-anti-miR-92a was tested in a catheter-based approach. The study compared systemic, retrograde, and anterograde delivery to the heart, all resulting in significant downregulation of miR-92a (155). MiR-92a appears to target mRNAs corresponding to several proteins in the context of angiogenesis, thereby leading to improved capillary density after ischemic challenge. The miR-92a family, including miR-25, miR-92a-1, miR-92a-2, and miR-363, originate from paralog clusters (miR-17-92, miR-106a-363, and miR-106b-25) that are highly conserved. The latter partly explains side effects that were observed during delivery of LNA-anti-miR-92a: miR-25 was downregulated just as miR-92a. Yet, other miRs of the miR-17-92a cluster were unaffected.

Despite local delivery, miR-92a levels were found to be affected in liver, kidney, and spleen, while the impact in lung tissue was lower compared to systemic delivery. However, significant decrease in infarct size and cell death as well as significantly improved global heart function were only observed after catheter-mediated anti-miR-92a delivery and not after systemic application (155). Furthermore, this study indicates that local delivery potentially enables reduction of anti-miR dose, thus resulting in reduced but not abrogated off-site effects (155). Again, this approach is in general feasible for targeting the vasculature but is less feasible for treatment of AAA since the compounds will be distributed with the circulation and are likely to accumulate in downstream capillary beds of non-target organs.

## DNA Origami for RNA delivery—an Outlook

While many revolutionary developments regarding RNA knowledge and technology have been mentioned in the sections above, this review will close with some short notes on a method that has just started to evolve in the DNA/RNA field and that might be promising in the context of AAA disease as well.

The DNA origami method is named after a famous Japanese paper folding art. Origami artists aim to sculpture a flat sheet of paper into a desired object by applying complex folding sequences. DNA origami aims to create a long single-stranded DNA molecule (scaffold) out of hundreds of short synthetic DNA nucleotides (staples), thereby folding specifically shaped objects at a nanoscale (156).

At first it was only possible to generate planar, two-dimensional objects (156), but since 2009 complicated three-dimensional structures can be effortlessly produced due to the introduction of novel design strategies (157). These powerful computational tools for designing and analyzing DNA nanostructures enable custom-made shapes (158) and lately even scaffold-free approaches (159, 160).

DNA-origami objects can load drugs and thereby function as miniature transporters. These so called “nanorobots” have been experimentally tested in the oncology field (161). But origami objects could (even more excitingly) also serve as miniature devices and, e.g., assemble critical molecules in cells, such as proteins and nucleic acids (162).

Although RNA molecules display many similarities to DNA molecules, it was not until 2013 that RNA origami had been reported by the Sugiyama and Mao groups (163, 164). In 2014, Sugiyama et al. (165) generated pure RNA origami objects by using RNA staples to fold the RNA scaffold. It is obvious that still a lot of research effort will be necessary to translate the RNA origami method into the cardiovascular field but it will be an exciting journey nevertheless.

## Conclusion

The RNA revolution has taken the modern research world by storm. It presented some major successes when it comes to discovering new biological paradigms and clinical-translatable technology. One disease that benefited from this extensive research effort—by reaching a better understanding of disease mechanisms—was AAA disease. Discovering underlying pathological mechanisms helped to outline solution strategies. Still, years of further research will be needed to take several technological hurdles and achieve the ultimate goal of clinical translation.

## Author Contributions

IS and AD drafted the manuscript. IS, UR, and PT contributed the initial idea, funding, and drafting. KM contributed ideas to the manuscript and created the figures. All authors contributed to the article and approved the submitted version.

## Conflict of Interest

IS and UR are cofounders of Angiolutions. Angiolutions is a company developing vascular devices for aneurysm diseases. The remaining authors declare that the research was conducted in the absence of any commercial or financial relationships that could be construed as a potential conflict of interest.
